# Supplementary figures and images for: Systematic comparison of unilamellar vesicles reveals that archaeal core lipid membranes are more permeable than bacterial membranes
Source: PLoS Biol. 2023 Apr 4;21(4):e3002048. doi: 10.1371/journal.pbio.3002048 (PMC10072491; doi:10.1371/journal.pbio.3002048)

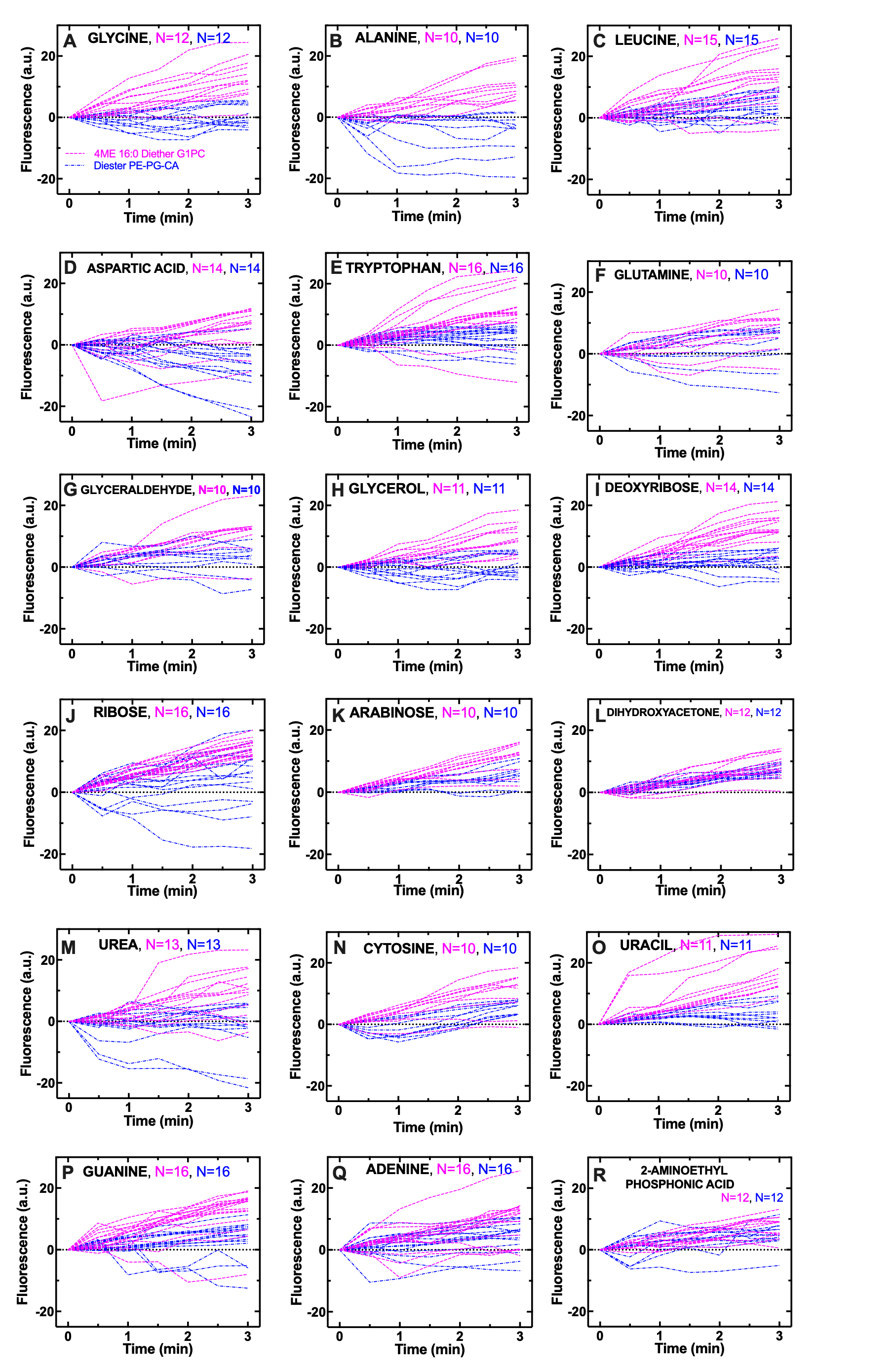

Supplement: S1 Fig — Temporal dependence of CF fluorescence in individual archaeal 4ME diether G1PC (magenta dashed lines) and bacterial diester G3PE-PG-CA vesicles (blue dashed-dotted lines) during the exposure to 1 mM of variant metabolites delivered to the microfluidic coves. N is the number of single vesicles investigated for each metabolite exposure and each type of vesicles (magenta and blue for archaeal 4ME diether G1PC and bacterial diester G3PE-PG-CA vesicles, respectively). N varies across different metabolite experiments investigated due to technical constraints (see Methods). However, care has been taken to obtain the same N for each metabolite experiment across the two different types of vesicles to ensure reliable statistical comparisons. The lipids used for creating archaeal 4ME diether G1PC and bacterial diester G3PE-PG-CA vesicles are lipids 1 and 2, respectively, in S1 Table. Numerical values of CF fluorescence in individual vesicles for each lipid type during the delivery of each metabolite are provided in Data A in S1 File. (TIFF) [file pbio.3002048.s001.tiff]

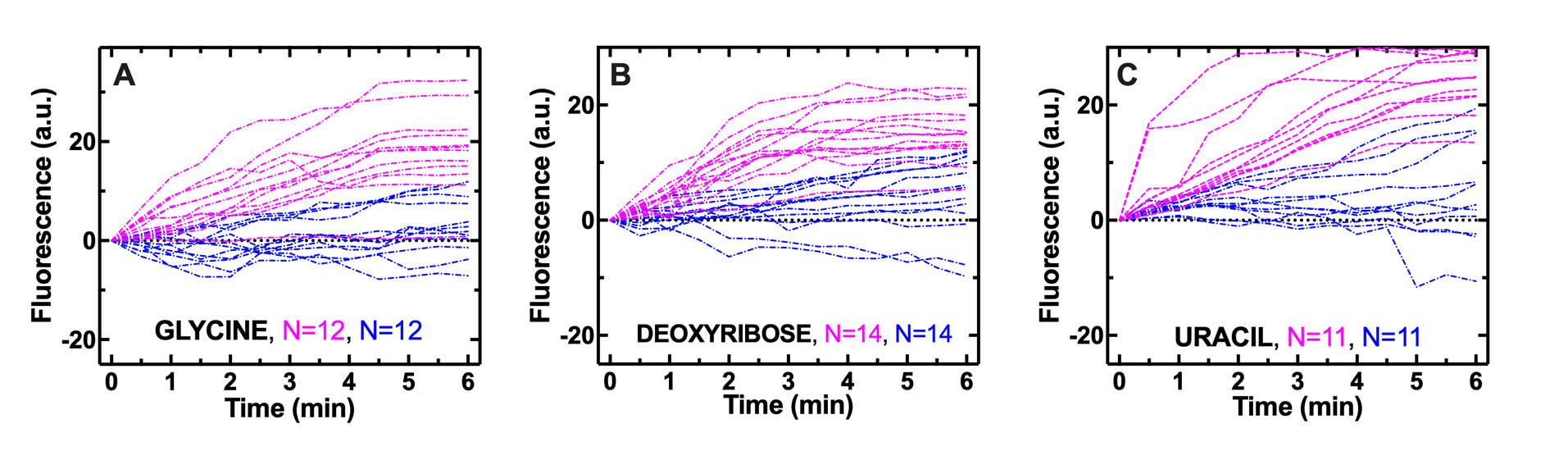

Supplement: S2 Fig — Temporal dependence of CF fluorescence in individual archaeal 4ME diether G1PC (magenta dashed lines) and bacterial diester G3PE-PG-CA vesicles (blue dashed-dotted lines) during the exposure to 1 mM of variant glycine, deoxyribose or uracil delivered to the microfluidic coves. Individual archaeal 4ME diether G1PC vesicles remain more permeable than bacterial diester G3PE-PG-CA vesicles over a 6 minute exposure to metabolites. Numerical values of CF fluorescence in individual vesicles for each lipid type during the delivery of each metabolite are provided in Data A in S1 File. (TIFF) [file pbio.3002048.s002.tiff]

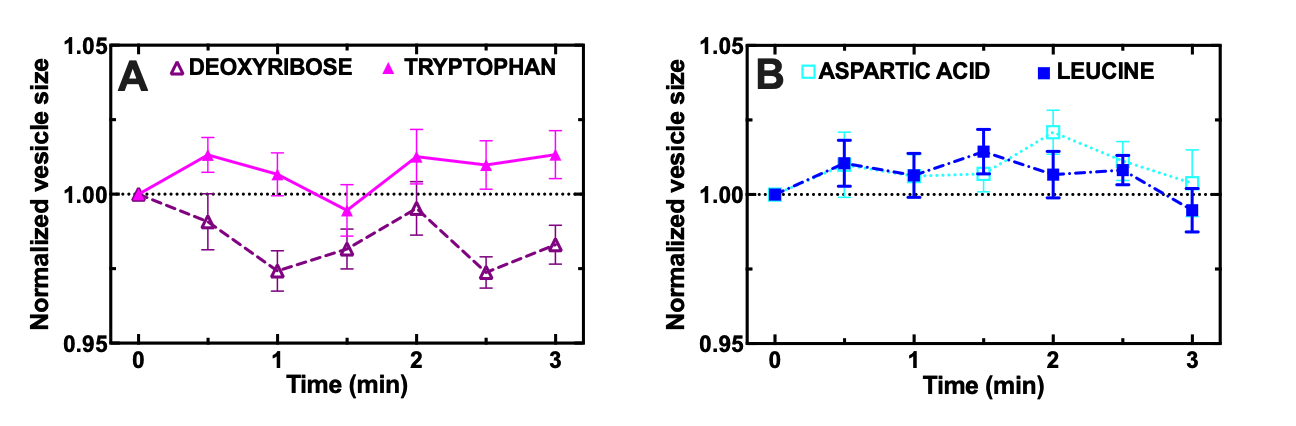

Supplement: S3 Fig — Temporal dependence of the average vesicle size during the delivery of 1 mM deoxyribose (open triangles) or tryptophan (filled triangles) to (A) archaeal 4ME diether G1PC vesicles and (B) leucine (filled squares) or aspartic acid (open squares) to bacterial diester G3PE-PG-CA vesicles. Mean (symbols) and standard deviation (error bars) were calculated by firstly normalising all values of each vesicle size to the size at t = 0 and then averaging over the values measured on N = 15 vesicles for each metabolite and membrane mimic. Corresponding permeability data for each metabolite and membrane mimic are presented in Fig 2. No significant change in vesicle size was measured during the delivery of deoxyribose or tryptophan to archaeal 4ME diether G1PC vesicles (p-value = 0.27 and 0.36, respectively) or during the delivery of aspartic acid or leucine to bacterial diester G3PE-PG-CA vesicles (p-value = 0.47 and 0.61, respectively). Moreover, no significant change in vesicle size or shape was measured during the delivery of any of the metabolites in Fig 2. The lipids used for creating archaeal 4ME diether G1PC vesicles and bacterial diester G3PE-PG-CA vesicles are lipids 1 and 2, respectively, in S1 Table. Numerical values of normalized vesicle size for each lipid type during the delivery of each metabolite are provided in Data A in S3 File. (TIFF) [file pbio.3002048.s003.tiff]

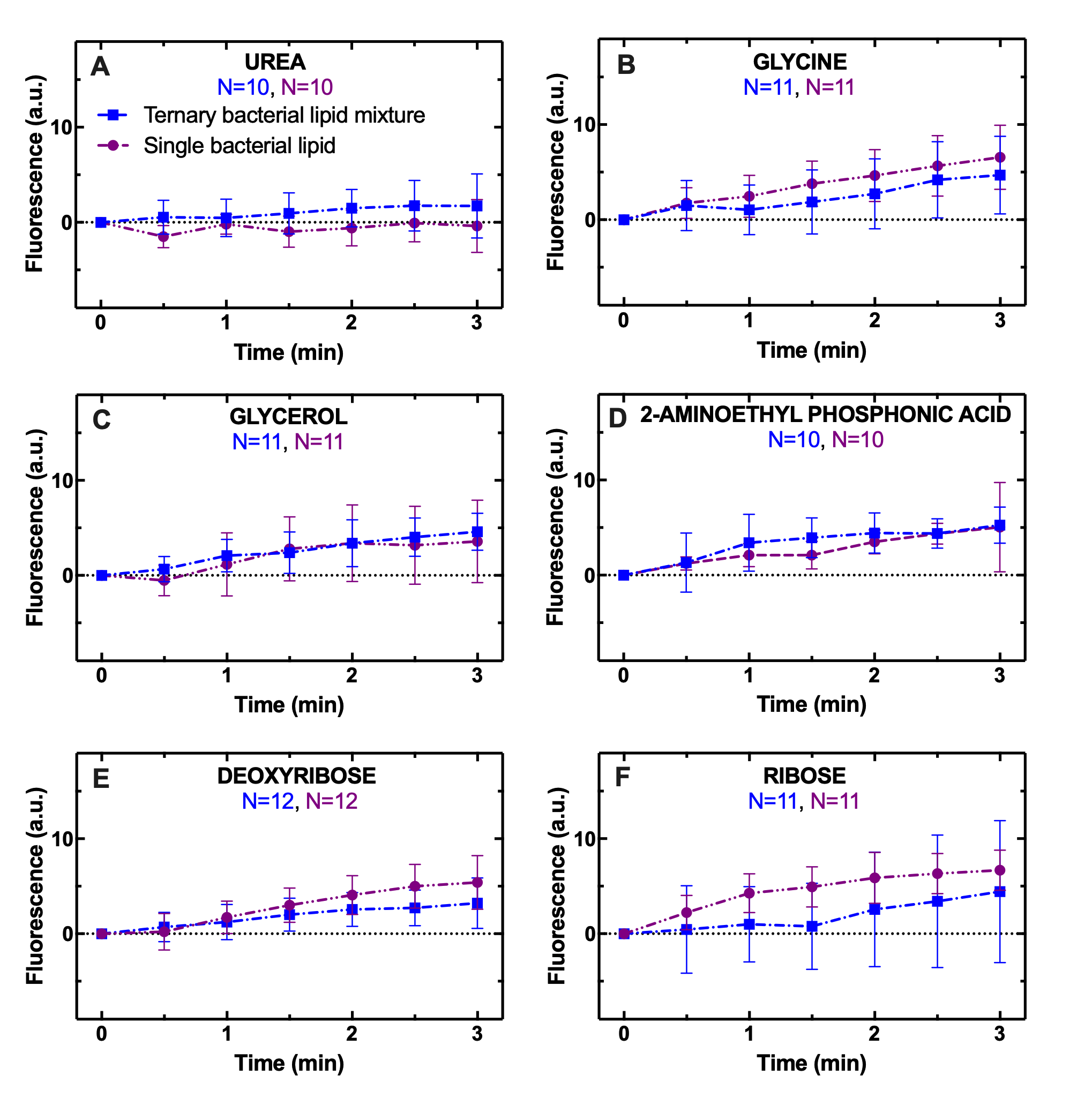

Supplement: S4 Fig — Temporal dependence of average CF fluorescence in bacterial vesicles made of one lipid (purple circles) or a ternary lipid mixture (blue squares) during the exposure to 1 mM (A) urea, (B) glycine, (C) glycerol, (D) 2-aminoethyl phosphonic acid, (E) deoxyribose, or (F) ribose delivered to the vesicles at t = 0. Mean (symbols) and standard deviation (error bars) were calculated from at least 10 single-vesicle measurements across 3 independent experiments. N is the number of single vesicles investigated for each metabolite and each type of bacterial vesicle (blue and purple for ternary and single lipid vesicle, respectively). N varies across different metabolite experiments investigated due to technical constraints (see Methods). However, care has been taken to obtain the same N for each metabolite experiment across the two different type of lipid vesicles to ensure reliable statistical comparisons. Such comparisons have been carried out via two-tailed Mann–Whitney tests between the distributions of CF fluorescence values at t = 3 min for the mono- and ternary-lipid mixture. The lipids used for creating the bacterial membrane mimics with ternary and single-lipid mixtures are lipids 2 and 6, respectively, in S1 Table. Numerical values of CF fluorescence in individual vesicles for each lipid type during the delivery of each metabolite are provided in Data B in S1 File. (TIFF) [file pbio.3002048.s004.tiff]

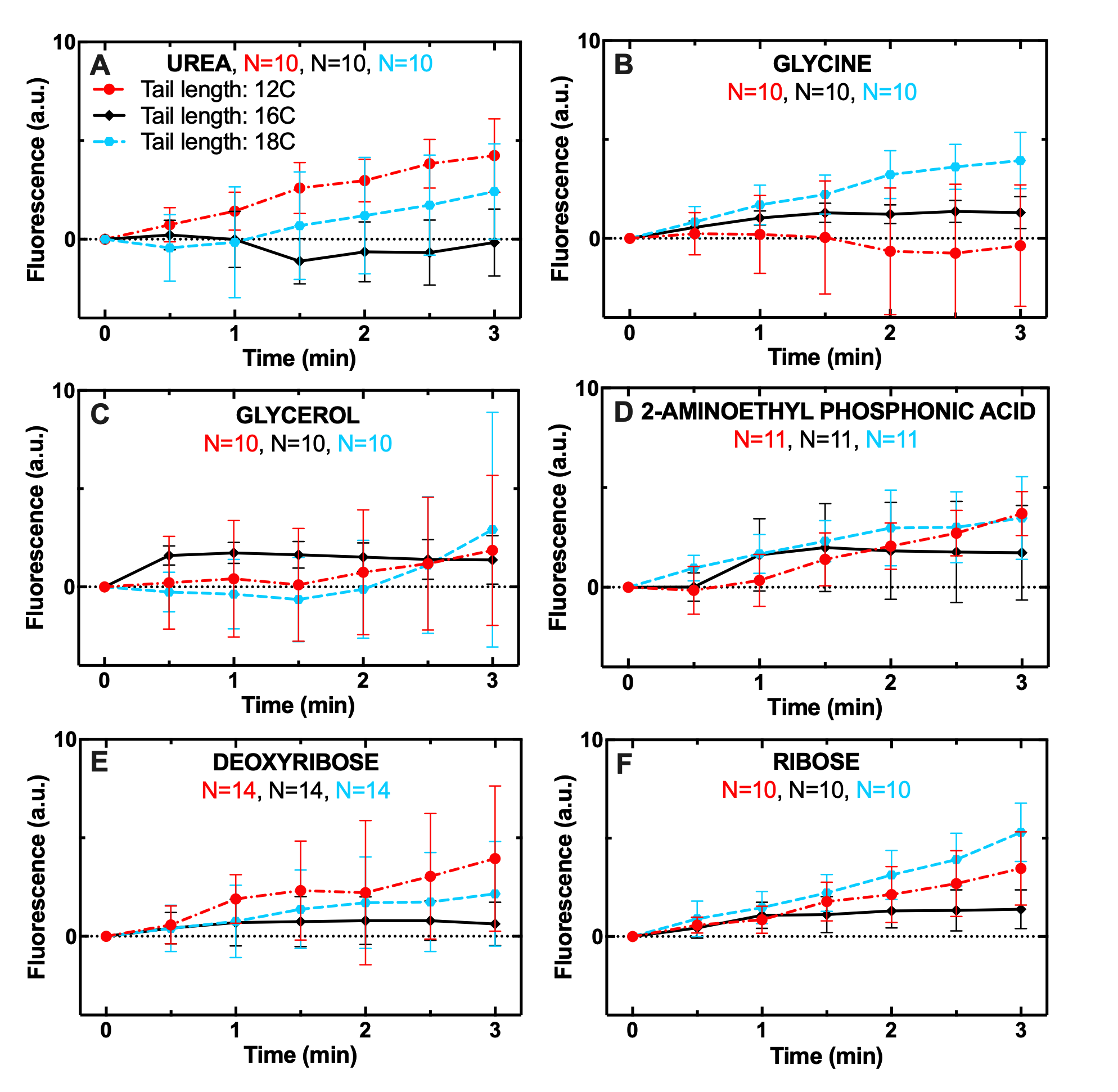

Supplement: S5 Fig — Temporal dependence of average CF fluorescence in lipids with tail length of 12 (red circles), 16 (black diamonds) or 18 carbons (blue hexagons) during the exposure to 1 mM (A) urea, (B) glycine, (C) glycerol, (D) 2-aminoethyl phosphonic acid, (E) deoxyribose, or (F) ribose delivered to the vesicles at t = 0. Mean (symbols) and standard deviation (error bars) were calculated from at least 10 single-vesicle measurements (solid lines) across 3 independent experiments. N is the number of single vesicles investigated for each metabolite and each lipid length. N varies across different metabolite experiments investigated due to technical constraints (see Methods). However, care has been taken to obtain the same N for each metabolite experiment across the three different lipid lengths to ensure reliable statistical comparisons. Such comparisons were carried out by evaluating the Pearson correlation coefficient between the average fluorescence value at t = 3 min and the tail length. The measured coefficients were −0.58, 0.95, 0.52, −0.10, −0.69, and 0.30 for urea, glycine, glycerol, 2-aminoethyl phosphonic acid, deoxyribose, and ribose and not statistically significant for any of these metabolites. We could not investigate the permeability of vesicles with chain length of 14 carbons (lipid 11 in S1 Table) because the transition temperature of these lipids (i.e., 24°C) is very close to room temperature and vesicles easily burst during our permeability assays. Finally, we could not form vesicles using lipids with a chain length of 6 carbons (lipid 10 in S1 Table) despite attempting different electroformation protocols (S3 Table). The lipids used for creating vesicles with tail length of 12, 16, and 18 carbons are lipids 8, 4, and 9, respectively, in S1 Table. Numerical values of CF fluorescence in individual vesicles for each lipid type during the delivery of each metabolite are provided in Data D in S1 File. (TIFF) [file pbio.3002048.s005.tiff]

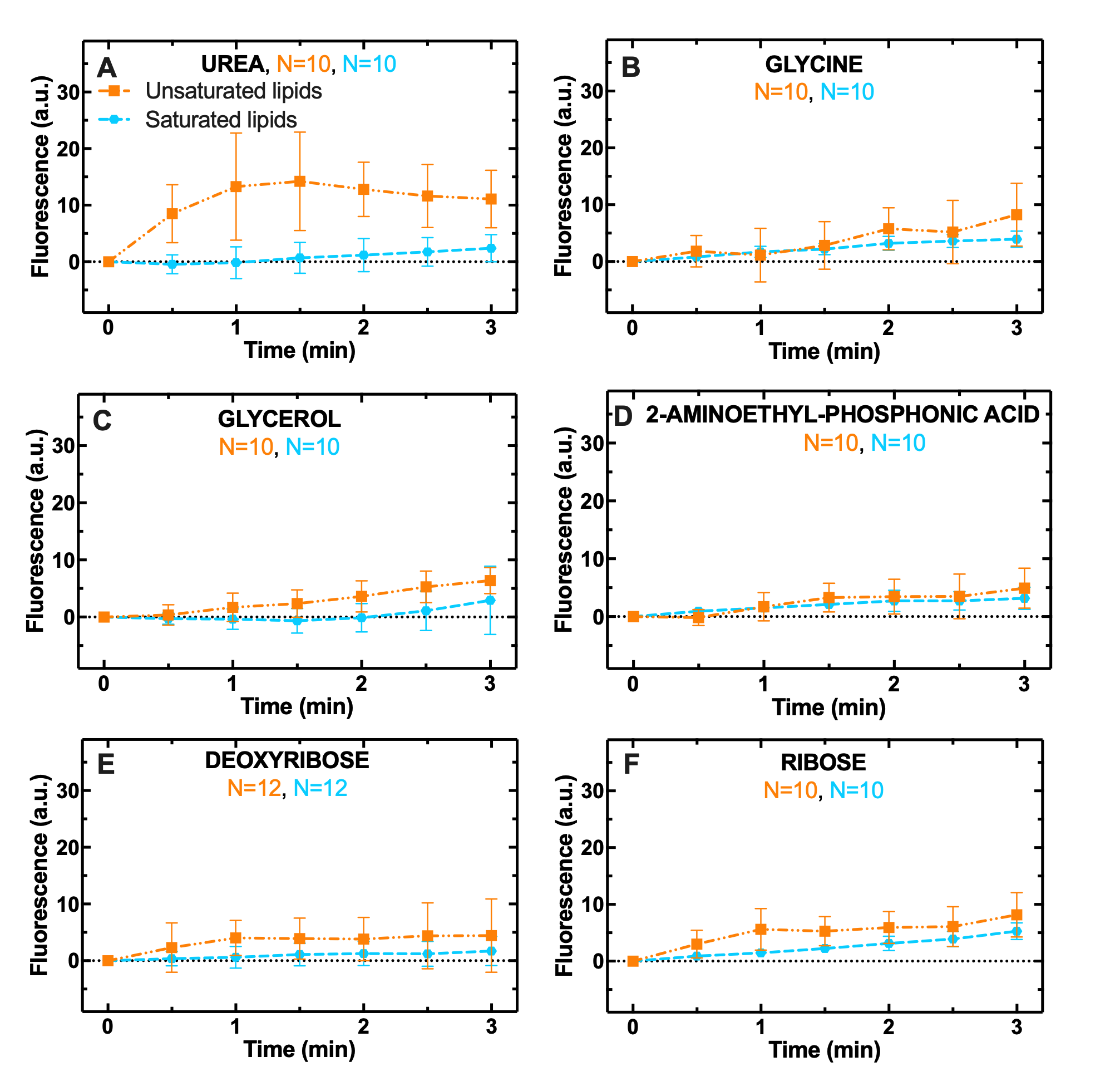

Supplement: S6 Fig — Temporal dependence of CF fluorescence in the presence (orange squares) or absence of double bonds along the lipid chain (blue circles) during the exposure to 1 mM (A) urea, (B) glycine, (C) glycerol, (D) 2-aminoethyl phosphonic acid, (E) deoxyribose, or (F) ribose delivered to the vesicles at t = 0. Mean (symbols) and standard deviation (error bars) were calculated from at least 10 single-vesicle measurements (solid lines) across 3 independent experiments. N is the number of single vesicles investigated for each metabolite and lipid type. N varies across different metabolite experiments investigated due to technical constraints (see Methods). However, care has been taken to obtain the same N for each metabolite experiment across the two different lipid types to ensure reliable statistical comparisons. Such comparisons have been carried out via two-tailed Mann–Whitney tests between the distributions of CF fluorescence values at t = 3 min for the single and double bonding lipid. The lipids used for creating the archaeal membrane mimics with and without saturation are lipids 9 and 12, respectively, in S1 Table. Numerical values of CF fluorescence in individual vesicles for each lipid type during the delivery of each metabolite are provided in Data E in S1 File. (TIFF) [file pbio.3002048.s006.tiff]

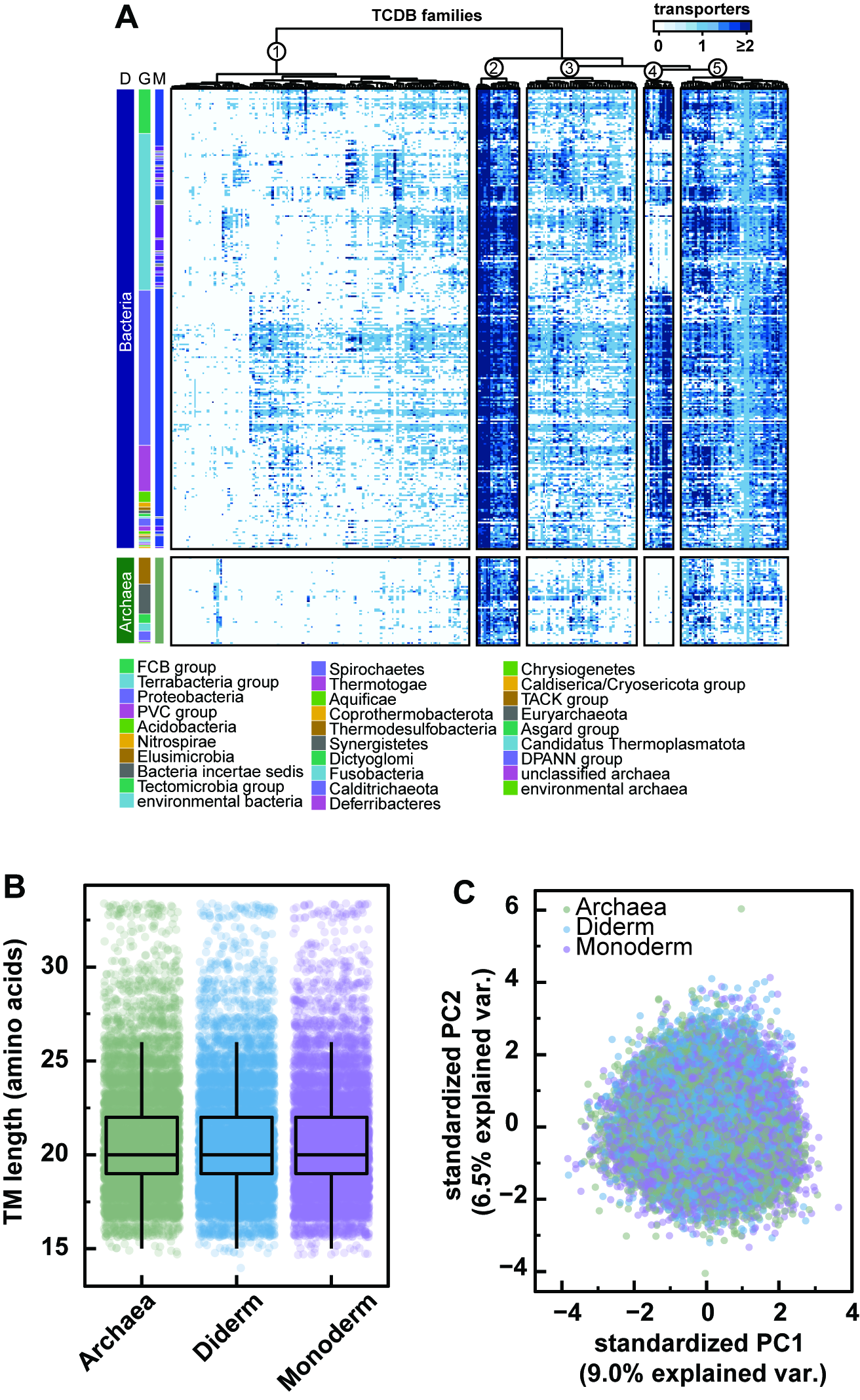

Supplement: S7 Fig — (A) Heatmap showing transporter repertoires in Archaea and Bacteria, where each row and column represent a prokaryotic order and TCDB transporter family, respectively. Heat map values represent the median number of transporters across each prokaryotic order. The domain (D), group (G), and membrane morphology (M: where blue, purple, and grey represent diderms, monoderms, and unknown membrane morphology, respectively) of each order is noted. TCDB families were grouped by hierarchical clustering based on transporter abundance using Euclidean distances and the Ward.D2 clustering method. Taxonomy was based on NCBI Taxonomy classifications. (B) Comparisons between the length of transmembrane domains in Archaea and bacterial monoderms and diderms. Transmembrane domains were identified using Phobius. (C) Principal component analyses based on the amino acid compositions of archaeal and bacterial transmembrane domains. In both analyses, archaeal and bacterial transmembrane domains were randomly subsampled to equivalent sample sizes (n = 10,000). Numerical values of the data presented are provided at https://doi.org/10.6084/m9.figshare.22086647. (TIF) [file pbio.3002048.s007.tif]
