# Supplementary material for: Systematic comparison of unilamellar vesicles reveals that archaeal core lipid membranes are more permeable than bacterial membranes
Source: PLoS Biol. 2023 Apr 4;21(4):e3002048. doi: 10.1371/journal.pbio.3002048 (PMC10072491; doi:10.1371/journal.pbio.3002048)
Supplement: S1 Table — List of lipids employed in this work, their chemical structure, their names according to the supplier (Avanti Polar Lipids within Merck), and their molecular weight. We could not produce vesicles using the lipids reported in grey due to technical limitations (see Methods). (DOCX) [file pbio.3002048.s008.docx]

| Lipid | Lipid chemical structure/ IUPAC name | Mimic type/Avanti name | MW [g/mol] |
| --- | --- | --- | --- |
| 1. | 1,2-di-O-phytanyl-sn-glycero-1-phosphocholine  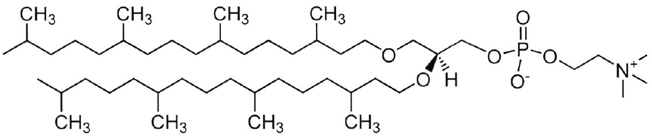 | Archaeal 4ME 16:0 Diether G1PC | 818 |
| 2. | 1,2-dioleoyl-sn-glycero-3-phosphoethanolamine  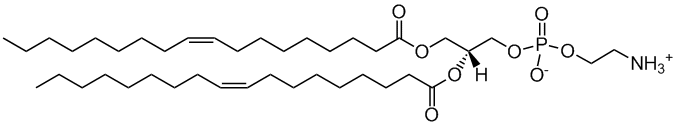  1,2-dioleoyl-sn-glycero-3-phospho-(1'-rac-glycerol)    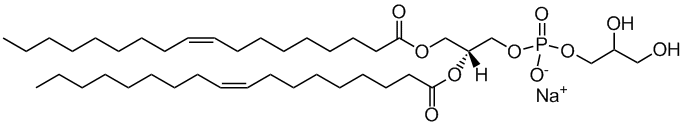    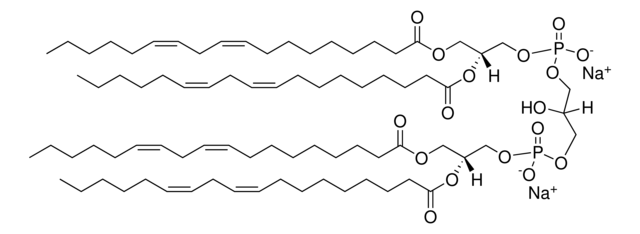 1,3‑*bis*(*sn*‑3'-phosphatidyl)-*sn*-glycerol | Bacterial Diester G3PE-PG-CA:67% : 18:1 (Δ9-Cis) Diester G3PE23.2% : 18:1 (Δ9-Cis) Diester G3PG 9.8% : Cardiolipin, sodium salt | 744    797  1494 |
| 3. | 1,2-di-O-phytanyl-sn-glycero-3-phosphocholine  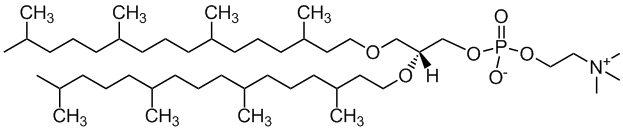 | 4ME 16:0 Diether G3PC | 818 |
| 4. | 1,2-di-O-hexadecyl-*sn*-glycero-3-phosphocholine 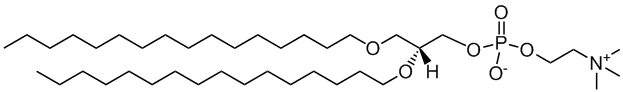 | 16:0 Diether G3PC | 706 |
| 5. | 1,2-diphytanoyl-sn-glycero-3-phosphocholine 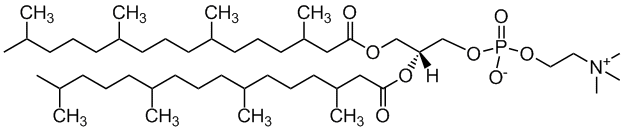 | 4ME 16:0 Diester G3PC | 846 |
| 6. | 1,2-dioleoyl-sn-glycero-3-phosphoethanolamine  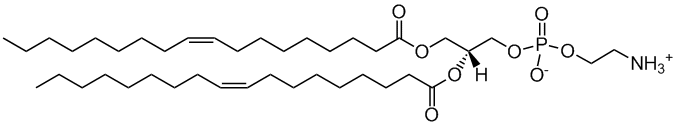 | Bacterial 18:1 (Δ9-Cis) Diester G3PE | 744 |
| 7. | 1,2-di-O-phytanyl-sn-glycero-3phosphoethanolamine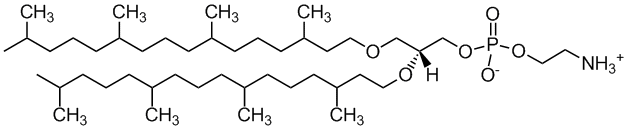 | 4ME 16:0 Diether G3PE | 776 |
| 8. | 1,2-di-O-dodecyl-sn-glycero-3-phosphocholine 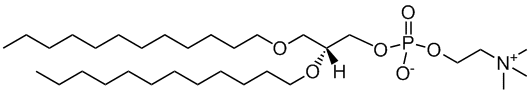 | 12:0 Diether G3PC | 594 |
| 9. | 1,2-di-O-octadecyl-sn-glycero-3-phosphocholine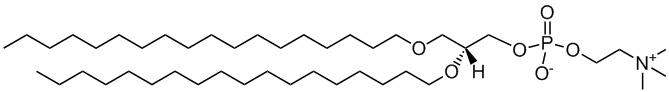 | 18:0 Diether G3PC | 762 |
| 10. | 1,2-di-O-hexyl-sn-glycero-3-phosphocholine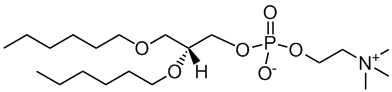 | 06:0 Diether G3PC | 426 |
| 11. | 1,2-di-O-tetradecyl-sn-glycero-3-phosphocholine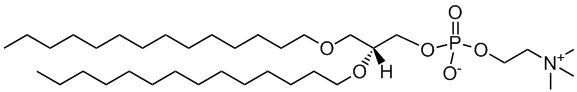 | 14:0 Diether G3PC | 650 |
| 12. | 1,2-di-O-(9Z-octadecenyl)-sn-glycero-3-phosphocholine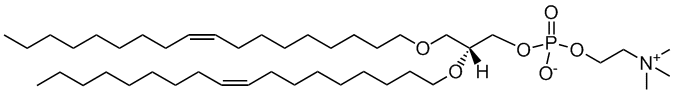 | 18:1 Diether G3PC | 758 |
