# Supplementary material for: Systematic comparison of unilamellar vesicles reveals that archaeal core lipid membranes are more permeable than bacterial membranes
Source: PLoS Biol. 2023 Apr 4;21(4):e3002048. doi: 10.1371/journal.pbio.3002048 (PMC10072491; doi:10.1371/journal.pbio.3002048)
Supplement: S2 Table — List of metabolites investigated in this work, the class they belong to, their molecular weight (MW), hydrophobicity (decreasing with XLogP3), their charge, the number of their rotatable bonds, the measured average fluorescence of the archaeal or bacterial lipid membrane mimic after 3-min exposure to each metabolite. (DOCX) [file pbio.3002048.s009.docx]

| Metabolite | Compound Class | MW  [g/mol] | XLogP3 | Charge | Rotatable  bond  counts | Fluorescence  at t=3min  Archaea | Fluorescence  at t=3min  Bacteria |
| --- | --- | --- | --- | --- | --- | --- | --- |
| Glycine | Amino acid | 75 | -3.2 | 0 | 1 | 13 | 1 |
| Alanine | Amino acid | 89 | -3.0 | 0 | 1 | 10 | -5 |
| Leucine | Amino acid | 131 | -1.5 | 0 | 3 | 12 | 4 |
| Aspartic Acid | Amino acid | 133 | -2.8 | -1 | 3 | 5 | -7 |
| Glutamine | Amino acid | 146 | -3.1 | 0 | 4 | 8 | 2 |
| Tryptophan | Amino acid | 204 | -1.1 | 0 | 3 | 11 | 2 |
| Glyceraldehyde | Sugar | 90 | -1.6 | 0 | 2 | 11 | 1 |
| Dihydroxyacetone | Sugar | 90 | -1.4 | 0 | 2 | 9 | 7 |
| Deoxyribose | Sugar | 134 | -2.3 | 0 | 4 | 13 | 2 |
| Ribose | Sugar | 150 | -2.5 | 0 | 0 | 15 | 7 |
| Arabinose | Sugar | 150 | -2.5 | 0 | 0 | 11 | 6 |
| Glycerol | Sugar alcohol | 92 | -1.8 | 0 | 2 | 10 | 5 |
| Cytosine | Nucleobase | 111 | -1.7 | 0 | 0 | 10 | 6 |
| Uracil | Nucleobase | 112 | -1.1 | 0 | 0 | 19 | 3 |
| Adenine | Nucleobase | 135 | -0.1 | 0 | 0 | 10 | 5 |
| Guanine | Nucleobase | 151 | -1.1 | 0 | 0 | 13 | 3 |
| Urea | Amide | 60 | -1.4 | 0 | 0 | 19 | 3 |
| (2-Aminoethyl)  phosphonic acid | Phosphonate | 125 | -4.8 | 0 | 2 | 8 | 5 |
| Adenosine monophosphate | Nucleotide | 347 | -3.5 | 0 | 4 | NA | NA |
