# Supplementary material for: Systematic comparison of unilamellar vesicles reveals that archaeal core lipid membranes are more permeable than bacterial membranes
Source: PLoS Biol. 2023 Apr 4;21(4):e3002048. doi: 10.1371/journal.pbio.3002048 (PMC10072491; doi:10.1371/journal.pbio.3002048)
Supplement: S3 Table — Parameters for the protocols employed in this work. The protocols reported in grey yielded a negative outcome (see Methods). (DOCX) [file pbio.3002048.s010.docx]

| Lipid | Frequency [Hz] | Amplitude  [V] | Temperature  [°C] | Time-rise  [min] | Time-main  [min] | Time-fall  [min] | Outcome |
| --- | --- | --- | --- | --- | --- | --- | --- |
| 1. | 5 | 3 | 37 | 5 | 120 | 5 | ✓ |
| 2. | 10 | 1.6 | 37 | 5 | 160 | 5 | ✓ |
| 3. | 5 | 3 | 37 | 5 | 120 | 5 | ✓ |
| 4. | 5 | 3 | 65 | 5 | 120 | 5 | ✓ |
| 5. | 5 | 3 | 37 | 5 | 120 | 5 | ✓ |
| 6. | 5 | 3 | 37 | 5 | 120 | 5 | X |
| 6. | 5 | 3 | 65 | 5 | 120 | 5 | X |
| 6. | 500 | 5.3 | 65 | 5 | 120 | 5 | X |
| 7. | 500 | 5.3 | 65 | 5 | 120 | 5 | ✓ |
| 8. | 500 | 5.3 | 65 | 5 | 120 | 5 | ✓ |
| 9. | 5 | 3 | 37 | 5 | 120 | 5 | X |
| 9. | 5 | 3 | 65 | 5 | 120 | 5 | X |
| 10. | 5 | 3 | 65 | 5 | 120 | 5 | X |
| 11. | 500 | 5.3 | 65 | 5 | 120 | 5 | ✓ |
