# Supplementary material for: Systematic comparison of unilamellar vesicles reveals that archaeal core lipid membranes are more permeable than bacterial membranes
Source: PLoS Biol. 2023 Apr 4;21(4):e3002048. doi: 10.1371/journal.pbio.3002048 (PMC10072491; doi:10.1371/journal.pbio.3002048)
Supplement: Attachment — Submitted filename: Lapinska et al-PLoS Biology-Response to Reviewers.docx [file pbio.3002048.s016.docx]

Responses to the revisions suggested by the Editor.

(a) *Please attend to the comments from the Academic Editor that can be found at the foot of this email. The Academic Editor (whose identity will be fairly self-evident) made it clear that while these are not mandatory, they might be advisable.*

We have attended to the comments made by the Academic Editor, please see below

(b) *The current Title will be very opaque to anyone outside the immediate field, and we ask that you change it to something much more explicit and informative. We suggest "Systematic comparison of unilamellar vesicles reveals that archaeal lipid membranes are more permeable than bacterial ones."*

We have changed the title as suggested

(c) *Please address my Data Policy requests below; specifically, we need you to supply the numerical values underlying Figs 2ABCDEFGHIJKLMNOPQR, 3ABCDEFG, 4AB, S1ABCDEFGHIJKLMNOPQR, S2ABC, S3AB, S4ABCDEF, S5ABCDEF, S6ABCDEF, S7ABC, either as a supplementary data file or as a permanent DOI’d deposition.*

The numerical values underlying all figures have now been provided in S1-S5 Files as requested.

(d) *Please cite the location of the data clearly in all relevant main and supplementary Figure legends, e.g. “The data underlying this Figure can be found in S1 Data” or “The data underlying this Figure can be found in* [*https://doi.org/XXXX”*](https://doi.org/XXXX%E2%80%9D)

The location of the data has been cited in the manuscript and in all relevant main and supplementary Figure legends.

Responses to the revisions suggested by the Academic Editor.

1. *On page 2 line 70 Introduction) the authors write: “An additional study has shown that, in the absence of counter-ions, liposomes made of diether lipids extracted from archaea (e.g. from Halobacterium salinarum) display lower permeability to protons compared to liposomes made of lipids extracted from bacteria”. I do not think it is possible to conduct a study without counter-ions: H+ has a charge and that charge must be balanced by something. That might be a Cl- ion. If an H+ crosses a membrane a positive charge moves into the cell, and that positive charge will oppose the influx of other H+ ions into the cell. If a Cl- also crosses into the cell, that positive charge will be partially dispersed by the negative charge. The rate at which H+ moves in therefore ultimately depends on the permeability of the membrane to Cl-, not H+. A counter-ion does not mean a positive charge moving the other way, but an ion that counters the build-up of charge, and that would include Cl-. It is therefore a counter-ion. If you used SO42- instead of Cl- and it crossed the membrane more slowly (which it probably would) then you would measure a lower permeability to protons. I have not read the papers cited but either they did not say what is imputed to them, or if they did then the authors did not know what they were talking about. Either way, this should be clarified for the authors not to look foolish to any bioenergeticist.*

We have now removed reference to counter-ions, on page 3 line 71.

2. *At the bottom of page 4 the authors write: “These data suggest that the metabolic selectivity of these membrane mimics is complex and does not rely solely on basic molecular properties, such as molecular weight or hydrophobicity.” As an aside, we have also found some strange patterns between hydrophobicity and binding interactions in Harrison et al (A biophysical basis for the emergence of the genetic code in protocells. BBA Bioenerg. 2022 1863: 148597)… It seems that a more subtle measure such as partition energy, discussed by in Caldararo & di Giulio (The genetic code is very close to a global optimum in a model of its origin taking into account both the partition energy of amino acids and their biosynthetic relationships, Biosystems 214 (2022), 104613). Some brief discussion of partition energy in terms of properties of metabolites crossing the membrane might be helpful.*

We have now discussed these suggested publications in the context of our findings on the correlation between membrane permeability and metabolite physicochemical properties on page 6 lines 196-206:

“Taken together with previous findings, demonstrating that solute hydrophobicity correlates poorly with permeability coefficients of eukaryotic fatty acid or phospholipid membranes [36] and that amino acid residue hydrophobicity might have not been key for the emergence of the genetic code [43], our data corroborate the hypothesis that subtle variations in the metabolite atomic structure could contribute to differences in membrane permeability [36,37,44]. In fact, we found a significant correlation between permeability and rotatable bond number for archaeal 4ME diether G1PC vesicles (Pearson coefficient r = -0.49, *), but not for bacterial diester G3PE-PG-CA vesicles (r = -0.32, non-significant at p = 0.05). It is also conceivable that other physicochemical properties such as the partition energy of amino acids (i.e. the propensity of amino acid residues to be exposed to water [45]), that has recently been proposed to be the most optimized property of amino acids in the genetic code [46], could contribute to differences in membrane permeability.”

3. *Page 9 bottom of page: The authors write: “Regardless, transporter reduction may have been facilitated, in part, by the increased metabolite permeability of archaeal lipid membranes which, in turn, reduces the requirement for, or utility of, transporter proteins.” This argument is interesting and may be true. But another possible explanation is simply differential loss of membrane proteins since then, as they are especially labile. See for example Sojo et al (Membrane Proteins Are Dramatically Less Conserved than Water-Soluble Proteins across the Tree of Life. MBE 33 (2016) 2874–2884). If, for example, archaea were more likely to be autotrophic than bacteria, then there may have been a greater tendency to lose membrane transporters as there was less need for them. Such a possibility might at least be mentioned.*

We have now acknowledged this possibility on page 12 lines 454-457: “However, it is also conceivable that if Archaea were more autotrophic than Bacteria, there may have been a greater tendency to lose membrane transporters in Archaea compared to Bacteria considering the increased propensity of transporter differential loss compared to water-soluble proteins [70].”

4. *Conclusions (page 10): “These are now admirably concise and give very little perspective. That might be a virtue, though a few extra words of context might not go amiss. I had previously specifically raised questions about the likelihood of the common ancestor of archaea being a heterotroph, and there are good recent papers from Williams et al and others on LACA and LBCA (which also imply autotrophic ancestry. Does this paper imply that archaea were heterotrophs, lived by recycling in biofilms, were autotrophs, or what? A few words that tentatively point to the significance of the paper in relation to what we think we might know from phylogenetics might not go amiss. Some of this was raised in response to the referees and could possibly be incorporated into the paper itself. I don’t need to agree; I would like them to offer something, even if couched tentatively.”*

Based on this suggestion. We have added a brief perspective on what these result mean for the origins of prokaryotes/cells debate, as suggested by the Editor.

“This selective permeability could potentially have provided the lineage that became the Archaea with access to specific metabolic resources without the need for a diversified cross-membrane transporter system. Such an evolutionary scenario suggests that the early archaeal forms and possibly early proto-prokaryotic forms with an archaeal-type core membrane (before the membrane transition, which marks the Archaea-Bacteria bifurcation and indeed the evolution of membrane transporters) possessed many heterotrophic functions (i.e. the ability to acquire chemical precursors for metabolic function via permeation from an external environment) [30,71,72]. Such a scenario does not exclude an early dependency on autotrophic functions at the origins of life [19,43,73,74] or indeed a metabolic network largely reliant on autotrophic function as the Archaea arose [9,61], nor does it exclude early phases of cellular evolution based on fatty acid membranes [30,75]. However, our results suggest as early life transitioned to prokaryote-type cells the acquisition of a proto-metabolic network within a prokaryotic membrane chassis could more readily be achieved within a core archaeal-type membrane chemistry. We argue that this is logical, because even if a central autotrophic proto-metabolism was entirely self-sufficient, as this system was expanded towards wider functions seen in LUCA, the ability to acquire and integrate additional metabolic resources, such as diversified nucleobases and amino acids, would be feasible in an archaeal chassis. This is because as proto-metabolic networks were compartmentalised and diversified within prokaryotic-like membranes, archaeal chassis possessed permeability to metabolic precursors, whereas bacterial type membranes did not.”
